# Comparison of the Effect of two Denture Cleansers on Tensile bond Strength of a Denture Liner

**Published:** 2013-09

**Authors:** M Farzin, F Bahrani, E Adelpour

**Affiliations:** a Dept. of Prosthodontics, School of Dentistry, Shiraz University of Medical Sciences, Shiraz, Iran.; b Dentist

**Keywords:** Denture Cleansers, Denture Liners, Tensile Strength

## Abstract

**Statement of Problem:** One of the most clinical challenging issues in prosthodontics is debonding of soft liners from the denture base.

**Purpose:** The aim of this study was to evaluate and compare tensile bond strength between soft liner and heat-cured acrylic resin when immersed in two different types of denture cleanser and distilled water, at different period of times.

**Materials and Method: **In this experimental in vivo study, 238 heat-cured acrylic blocks were made. A soft liner was embedded between the acrylic blocks. Samples were divided into four groups: 17 samples were in the control group and were not soaked in any solution .The remaining samples were divided into 3 groups (Distilled water, Calgon and Fittydent). Each group was then subdivided into two subcategories, regarding the immersion time variable; 15 and 45 minutes. All samples were placed in tension force and tensile bond strength was recorded with the testing machine. One- way ANOVA and Tucky HSD post-hoc test were adopted to analyze the yielded data (α> 0.05).

**Results: **Specimens which were immersed in two denture cleansers (Fittydent and Calgon) and in distilled water showed significant difference (*p*= 0.001) in bonding strength when compared to the control group. The subjects immersed in denture cleanser solutions and distilled water did not reveal any significant difference (*p*= 0.90). For all groups; most of the bonding failures (72%) were cohesive type.

**Conclusion: **The effect of the denture cleansers and distilled water on the bond strength was not statistically different; however, the difference was significant between the immersed groups with the non-immersed group. Moreover, type of the denture cleanser did not show any effect on the tensile strength. The tensile strength increases with time of immersion.

## Introduction

When patients cannot bear the hard denture base; denture soft lining (DSL) materials are employed to replace the intaglio (interior) surface of a conventional hard denture to achieve an equal force distribution, to reduce confined local pressures and to improve retention of an ill-fitting denture by involving the undercuts [1-2]. 

The most favorable properties of soft liners are their resiliency which makes them absorb the mastication forces and disperse this impact force over the involved alveolar ridge. Therefore; the consequent mucosal pain may be relieved and the patient’s masticatory efficacy, biting force and chewing habits will be improved [3-4]. 

Denture soft liners (DSLs) have been introduced to dentistry for more than a century. The earliest DSLs were made from natural rubber and probably the first synthetic soft liner resin adopted in 1945 was a plasti- cized polyvinyl resin [1, 4]. In 1958, silicon-based materials were presented [5].

**Table 1:** Materials used in the study

**Product**		**Composition**	**Manufacture**
Resilient liners Ever soft	Powder: Polyethyl methacrylate, Liquid: dibutyl phthalate, ethylalcohol, ethyl acetate.		Myerson,Austenal,Inc, Chicago, USA
Denture base Resin	Powder: methyl metacrylate ethacrylate, dibutyl paleoteodine, benzoyl peroxide Liquid: methyl methacrylate, tophanol, ethylene glycol, di-methacrylate		Bayer-Germany
Denture cleanser Fitty dent	Sodium perborate,Potassium mono sulfate (PEG-240), sodium bicarbonate.		Fittydent International, GmbH, Austria
Denture cleanser Calgon / Clorox	Sodium hypochlorite, Sodium chloride, water, sodium salts: hexametaphosphate, chloride, carbonate, bicarbonate, triphosphate		The Clorox Co., Oakland, Beecham Products, Pittsburgh, PA, USA

These soft lining materials can be divided into acrylic-and silicon-based groups and both groups are offered in auto-or heat-cured systems. Auto-cured methods are used directly in the mouth and are faster than heat- cured systems but providing the best thickness with the auto-cured method is challenging [1, 4]. 

Acrylic-based soft lining materials usually are comprised of powder and liquid components. The composition of the powders and liquids are mostly considered to be methacrylate polymers and copolymers, along with a liquid containing methacrylate monomer and plasticizers (ethyl alcohol and/or phthalate) [1, 6].

When these materials are immersed in water; two processes are feasible: The plasticizers and other soluble constituents leach into the water and the polymer absorbs the water which, with time, leads to the change in the physical and mechanical properties of the materials in the patient’s mouth [3]. The leaching out of the plasticizer may result in the loss of resiliency and [7] and changes in the visco-elastic properties of DSLs so that they become hard and brittle and lose their bond strength properties [8]. 

The use of DSLs are accompanied by several problems such as the failure of bonding between the soft liner and the denture base, loss of resiliency, color alterations, formation of porosity and consequent plaque accumulation and Candida albicans colonization, and poor tear strength [4, 6]. 

Bond failure, one of the most serious problems, provides a potential surface for bacterial growing, plaque and calculus formation [4, 9]. 

The absence of proper bonding to the denture base materials will overcome other virtuous properties of DSLs. Many factors affect the bond strength between the DSL and the denture base, including water absorption, surface primer usage, and denture base composition [10]. The increased porosity of DSLs in their clinical use may lead to the accumulation of plaque and colonization of Candida albicans [11]. To prevent the consequent denture stomatitis; two methods are employed: mechanical plaque control (most likely) and chemical plaque control [1, 4]. 

Brushing is probably not advisable because it can damage the resilient lining [4]. The immersion with chemical agents is primarily the preferred method for geriatric patients and for patients with poor motor-nerve capabilities [12]. 

The chemical solutions used for denture cleansing can be divided based on their chemical components such as alkaline peroxide solutions; hypochlorite solutions, acidic solutions, disinfectants, and enzymatic solution that are more effective in precluding microbial invasion and plaque accumulation [4, 13]. 

Denture cleansers, nevertheless, can cause substantial deterioration since they can similarly cause loss of soluble components and plasticizers and as the consequence, DSLs can absorb water or saliva [13-14]; a process which can impact the properties of these materials.

The proper selection of denture cleanser is then crucial to avoid or minimize any plausible alterations in the properties of DSLs [1, 4, 14]. 

This study was conducted to evaluate changes in tensile bond strength of one resilient liner bonded to heat-cured acrylic resin when immersed in two denture cleansers and 37°C distilled water.

## Materials and Method

The DSL materials, denture base acrylic resin and the denture cleansers used in this in vitro study are presented in Table 1. These materials were selected for the current study since they appear to be more successful in clinical use compared to other brands.

Total of 238 specimens (blocks) of heat-cured polymethyl methacrylate (PMMA) with 20 mm in length, 20 mm in width and 10 mm in thickness were prepared. Two PMMA plates were prepared by investing brass dies with a 4- mm- thick spacer in a laboratory denture flask. All the dies and spacers were calibrated to the same proportions to standardize the shape of the denture base samples and the thickness of the DSLs.

The dies and spacer were invested in hard, but flexible silicon rubber (Lastic Xtra; Kettenbach, Germany) to ease the removal of the processed specimens from the flask. Specimens were made by processing the DSL against polymerized PMMA blocks. DSL and heat- polymerized acrylic resin were processed according to the manufacturers' recommendations. After processing the acrylic blocks, the 2 polymerized PMMA specimens were removed from the flask. The edges of the blocks were trimmed using a # 300 silicon-carbide abrasive paper (Norton; Sao Paulo, Brazil). The brass spacers were then removed from the denture flask. The PMMA blocks were supplanted in the mold and the denture soft liner was packed in to the space left by the brass spacers and then polymerized. Totally 119 specimens were prepared with two blocks of heat- cured acrylic resin bonded together by a 4- mm- thick layer of soft liner materials, and then the flasks were placed under standard pressure (No.01001;Teledyne Hanau, Buffalo, NY) for 15 minutes. The specimens were randomly divided into 4 groups. The first group consisted of 34 specimens immersed in 37°C distilled water in a way that 17 blocks were immersed for 15 days (D_15_) and 17 blocks were immersed for 45 days (D_45_). The second group consisted of 34 specimens which were immersed in the Fitty dent solution for 30 minutes at 22±2°C once in 24 hours, washed thoroughly under tap water and stored in distilled water for the rest of the day at 37°C; 17samples were undergoing the same procedure for 15 days (F_15_) and 17 samples for 45 (F_45_) days. The third group consisted of 34 specimens immersed in Calgon/Clorox solution for 8 hours at 22±2°c once in a day based on the manufacturer’s recommendations, washed thoroughly with tap water and stored in distilled water. They were then immersed into distilled water at 37°C for the remaining 24 hours; 17 specimens have been through the same procedure for 15 days (C_15_) and the other 17 specimens for 45 days (C_45_). For each immersion, fresh solutions of the denture cleansers were provided concerning the manufacturer’s recommendations and the distilled water was changed daily. The fourth group consisted of 17 specimens that were tested immediately after soft liner curing without immersion (W). Table 2 represents the treatments which were carried out on each group. Tensile bond strength was determined with a universal testing machine (EMIC DL 500MF; Equipamentose sistemas de Ensaios LTda, Parana, Brazil). The specimens were placed under tension until failure; using a crosshead speed of 5 mm /min until fracture occurred.

**Table 2:** The treatment employed for each group

**Group**	**Kind of treatment**	**N**	**Mean ±** **Std. Deviation**
D_15_	Immersed in distilled water for 15 days	17	4.77±0.67
D_45_	Immersed in distilled water for 45 days	17	8.24±0.52
F_15_	Immersed in Fittydent for 15 days	17	5.10±0.61
F_45_	Immersed in Fittydent for 15 days	17	8.88±0.59
C_15_	Immersed in Calgon for 15 day	17	5.99±0.24
C_45_	Immersed in Calgon for 45 day	17	9.98±0.28
W	Without immersion	17	1.58±0.35

The load at which the bonding failure occurred was recorded. The type of failure (Table 3) was observed using a stereoscopic microscope (Corl Zeiss, Gottingen, Germany) at original magnification of 8X. The failures that occurred at the soft liner-acrylic resin interface were recorded as “adhesive”, and those failures that occurred within the soft liner were considered to be “cohesive”. 

**Table 3:** Mode of failure in four groups

**Material **	**Cohesive**	**Adhesive**	**Mixed**
Distilled water (D)	%80	%3	%17
Fittydent (F)	%73	%2	%25
Calgon (C)	%75	%5	%20
Without immersion (W)	%60	%2	%38
Total	%72	%3	%25

An adhesive/cohesive failure was recorded when there were no complete separations between the materials. One- way ANOVA and Tukey HSD post-hoc tests were adopted to analyze the obtained data (α= 0.05). For all statistical analysis, the significance level was set at *p*< 0.05.

## Results

The mean and the standard deviations of tensile bond strength are listed in Table 2. The specimens which were immersed in distilled water and denture cleansers (Fitty dent and Calgon) showed a significantly higher tensile bond strength than the group without immersion (*p*= 0.001). Comparison of the treatments showed no significant difference between two cleansers and distilled water (*p*= 0.90), but those specimens immersed in Calgon/Clorox exhibited higher tensile strength but not statistically different (Table 4).

**Table 4:** The results of the statistical analysis

**Material**		**Mean ** **difference**	**Std. ** **Error**	**Significant**
Distilled water	Fittydent	-.4864	70876	.902
	Calgon	-1.4800	70876	.173
	Without immersion	4.9250	86805	.000*
Fittydent (F)	Distilled water	.4864	70876	.902
	Calgon	-.9936	70876	.505
	Without immersion	5.4114	86805	.000*
Calgon (C)	Water	1.4800	70876	.173
	Fittydent	.9936	70876	.505
	Without immersion	6.4050	86805	.000*
Without Immersion (W)	Distilled water	-4.9250	86805	.000*
	Calgon	-5.4114	86805	.000*
	Fittydent	-6.4050	86805	.000*

*These findings are statistically significant (*p*< 0.05

**Figure 1:**
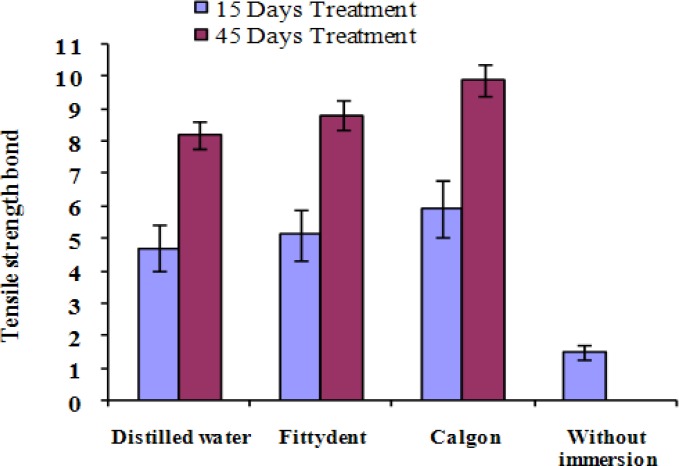
Tensile bond strength according to the employed treatment in each period of time

When specimens were immersed in distilled water, Fitty dent and Calgon/ Clorox, the data were significantly different (*p*< 0.001) with time, with the highest values varying from 1.58±0.35 to 9.98±0.28 MPa (Figure 1). For all groups, most of the failures (72%) were cohesive for the studies of liner material (Table 3).

## Discussion

Proper denture hygiene is always imperative and many patients who wear denture do not have an acceptable level of hygiene [15]. Therefore, an inclusive range of denture cleansers are provided to develop denture hygiene. Daily usage of denture cleansers can influence the physical properties of acrylic denture bases and soft liners [4]. 

In this study, the effect of two denture cleansers on tensile bond strength of a soft liner was evaluated. The results showed that tensile bond strength increased with time for both denture cleansers (Fitty dent and Calgon) and distilled water. The absorption or loss of soluble constituents of DSLs may cause failure in bond strength between the DSL and denture acrylic resin of the denture base [1, 6]. The results of the current study are in agreement with Gracia et al. [8], Sinobad et al. [10], Hong et al. [16] and those of others, who proposed that water immersion increased the bond strength of soft liners [8, 15]. This might have occurred as a result of leaching out of the plasticizer which in turn leads to the increased stiffness and hardness [17]. 

A direct comparison of studies cannot be made because of the different study methods which were employed in different studies. In the Meşe study [4], the comparison between Polident and water immersion showed no variation in the tensile bond strength of the four studied soft liners for the trialed time periods. These findings were in line with the study of Garcia et al. [8] who reported specimens immersed in Polident compared with water; tensile bond strength was not altered. Regarding the study of Kazanji et al. [18] soft lining material can absorb water or lose soluble components based on their structure and the chemical solution in which they are soaked. The authors believe that the higher ionic concentration of denture cleanser such as Potassium and Sodium resulted in a higher release of soluble constituents when these cleansers are compared with water. In this study, the specimens immersed in Calgon/Clorox showed the highest tensile bond strength with time in comparison to other study groups. However, the values obtained in this study were very high when compared with those recorded by Mese et al. [1] who stored the soft liners in the water. These different results are likely due to the different study protocols. The current study showed that for studied soft liner (Ever soft) most failures were classified as cohesive (72%) which can be explained by the mechanical bonding and the chemical adhesion that happened. Mechanical bonding occurred because of the sandpaper treatment of the acrylic resin surface which consequently may increase the surface roughness and mechanical retention. Chemical adhesion might be due to the comparable chemical composition of the acrylic resin and the soft liner [10, 19]. The selection of denture cleanser depends on many factors and since these chemical solutions can cause substantial deterioration on the soft liners, compatible materials should be considered to avoid or minimize any changes in the favorable physical or chemical properties [20]. Future In vivo studies are recommended if the results of the current and other previous studies are clinically applicable.

## Conclusion

Specimens immersed in denture cleansers and distilled water demonstrated increased tensile bond strength with time. The effect of the denture cleansers and distilled water on the bond strength was not statistically different; however, the difference was significant between the immersed groups with the non-immersed group. Moreover, type of the denture cleanser did not show any effect on the tensile strength. The tensile strength increases with time of immersion.
